# An Assessment of the Consumption of Energy and Selected Minerals and Their Content in the Hair of Children Aged 1–4 Years

**DOI:** 10.1007/s12011-015-0469-2

**Published:** 2015-08-21

**Authors:** Katarzyna Marcinek, Rafał Wojciech Wójciak, Zbigniew Krejpcio, Maia Stanisławska-Kubiak

**Affiliations:** Department of Human Nutrition and Hygiene, Poznań University of Life Sciences, Poznań, Poland; Department of Clinical Psychology, Poznań University of Medical Sciences, Poznań, Poland

**Keywords:** Children, Trace element, Hair, Daily food rations, Mineral content

## Abstract

The aim of this study was to assess the consumption of energy and selected minerals (Ca, Mg, Fe, Zn, Cu) and their content in the hair of children aged 1–4 years. Seventy-five children were divided into three age subgroups: 12–24-month-olds, 25–36-month-olds and 37–48-month-olds. The data on energy intake and consumption of nutrients were obtained by means of a nutritional interview. The content of elements in the hair was measured by means of flame atomic absorption spectrometry (AAS) with an AAS-3 spectrophotometer (Zeiss). The daily food rations of children aged 12–36 months were characterised by adequate energy value, but the values of Ca and K were too low, whereas the content of Mg, Zn and Cu was high. On the other hand, the daily food rations of children aged 37–48 months provided sufficient amounts of Mg and Zn, but the value of Cu was too high, whereas energy, Ca, K and Fe were too low. About 42.7 % of the children under study were characterised by an abnormal state of nutrition. An inadequately balanced diet needs to be corrected by educating parents or guardians in appropriate nutrition. There are significant correlations (*r* > 0.9) between the supply of Ca in the diet of children aged 1–4 years and the content of this element in their hair.

## Introduction

Adequate nutrition is one of the most important environmental factors with favourable influence on children’s health and their learning capacity, motoric activity, well-being and emotional states. Children are a group of people which is the most sensitive to mistakes in nutrition. At this age, we can observe unfavourable changes in the somatic development caused by a badly balanced diet. The manner of nutrition in childhood exerts significant influence on people’s nutrition in adulthood, and eating habits from childhood often affect later choices [1]. Inadequate eating habits may cause the development of numerous diseases, such as obesity, type 2 diabetes, hypertension, osteopenia or osteoporosis. They also increase the risk of dental caries, anaemia and cardiovascular diseases, and they delay growth and maturation. The first 3 years in childhood are a period of particularly intensive physical, intellectual and motoric development. During this period, the manner of children’s nutrition changes from the one that is typical of infanthood to the manner of nutrition which is typical of adults [2]. Adequate balance of energy and supply of minerals is particularly important in early childhood. A long-lasting deficit, excess or inadequate balance of nutrients may have serious consequences in the form of diseases that are typical of a particular nutrient or disorders leading to the development of diet-dependent diseases [3]. Minerals should be provided in adequate amounts and mutual proportions due to their role in metabolism and due to the fact that they cannot be synthesised by the organism. Studies which have been published so far mostly concentrate on the evaluation of nutrition in nursery schools and other catering outlets [4]. Data on the nutritional value of daily food rations of children are still incomplete [5]. In view of the important role of public institutions and parents in developing feeding habits, the aim of this study was an assessment of the supply of energy and minerals in daily food rations (DFRs) of children aged 1–4 years fed at home and in nursery schools. Another aim of this study was an assessment of the content of minerals based on the analysis of the content of elements in children’s hair.

## Material and Methods

The Bioethical Commission, Medical University of Poznań, gave the permission for the study (no. 871/10). The study was conducted in 75 children aged 1–4 years living in Poznan and the surrounding area. The population was divided into three age subgroups: 12–24-month-olds (27 children), 25–36-month-olds (19 children) and 37–48-month-olds (29 children). The first two subgroups consisted of children staying at home with their parents or legal guardians. Children attending nursery schools were recruited to the subgroup aged 37–48 months. The children’s age was the criterion for inclusion into the group under study (12–48 months), whereas the exclusion criterion was the children’s illnesses, which caused the need to apply a different method of nutrition than oral nutrition. The study was conducted in April 2014.

### Anthropometric Test

Anthropometric measurements were made according to the generally accepted methodology, in the morning, without outerwear and shoes. The height and body weight were measured on medical scales with an accuracy of 0.10 cm (body height) and 0.10 kg (body weight). The anthropometric data gathered in the research were used to calculate the body mass index (BMI) values (kg/m^2^). Then, they were standardised with reference to the WHO growth charts. For each child, a standardised body mass index *z*-score and percentile values of the body weight, body height and body mass index were calculated [6].

### Assessment of Energy Intake and Consumption of Nutrients in DFR

The assessment of the children’s method of nutrition was based on 24-h records of the 75 children’s menus, which their guardians made by means of current note taking for seven consecutive days. The data on the amount of consumed portions were obtained by weighing ready meals and leavings. The data gathered in this way were used to estimate the DFR, and its energetic and nutritional value was calculated by means of the ‘Dietetic 2’ nutritional computer program. The calculations allowed for culinary and technological losses of 10 %.

### Elemental Analysis of Hair

About 0.3 g of hair samples were collected from six different spots in the occipital region of the head. Pieces of hair, which were about 1 cm long, were cut right next to the skin. The hair samples were washed in acetone and deionised water, and then, they were dried to a constant weight at a temperature of 105–110 °C. The dry hair samples were weighed and mineralised (with 5 cm^3^ of concentrated HNO_3_, Merck) in a Mars 5X mineraliser (CEM, USA) with pressure (ESP-1500 Plus) and temperature control systems (RTP-300 Plus). Next, the acidic mineralisate was quantitatively transferred with deionised water into 10-ml volumetric flasks for quantitative measurement of elements. The content of elements in the mineralisates was measured by means of flame atomic absorption spectrometry (AAS) with an AAS-3 spectrophotometer (Zeiss). The results were expressed as the mean ± SD in microgrammes/gramme of dry weight. The correctness of the analytic procedure was determined by means of the recovery volume of minerals for certified reference material (human hair, 397). For Ca, Mg, Fe, Zn and Cu, the values of 98.2, 96.6, 96.7, 94.2 and 98.5 % were recorded, respectively.

### Statistical Analysis

This study assumed a 15 % standard error in the estimation of the values of the nutritional indexes under investigation, and the minimum size of the population subgroup calculated for the error was 25 people. Microsoft Excel 2007 and Statistica 10.0 Pl programs were used for all calculations. The normality of distribution was checked by means of the Shapiro-Wilk test. The one-way analysis of variance and Tukey’s test were used to determine age-dependent differences in the children’s consumption of energy and minerals. Sex-dependent differences in the children’s consumption of energy and minerals were determined by means of Student’s *t* test. Pearson’s correlation coefficients were calculated to assess the dependence between the consumption of individual bioelements in the DFR and their content in the hair. The results were analysed at a significance level *p* = 0.05.

## Results

Table 1 shows the characteristics of the group of children under study. Each child’s state of nutrition was assessed by means of sex-and-age-independent body mass index *z* score. Of the children, 57.3 % were in a normal state of nutrition and their BMI *z*-score ranged from −1.0 to +1.0. Low body weight (BMI *z*-score between −2.0 and −1.0) was observed in 17.3 % of the children, whereas very low body weight (BMI *z*-score <−2.0) was observed in 4.0 % of the children. Overweight and obesity were observed in 20.0 and 1.3 % of the children, respectively. The children aged 37–48 months were characterised by much lower WA (weight-for-age) and LA (length/height-for-age) than the younger children. Tables 2 and 3 show the data about the intake of energy and minerals in the children’s DFRs with reference to the recommended dietary allowance (RDA; for energy % energy efficiency rating (EER) and for Na, K % AI were given) depending on the children’s age and sex. The data revealed that the intake of energy was comparable in the DFRs of 12–24-month-old children and 25–36-month-olds, and it satisfied about 100 % of the demand (EER), in contrast to the DFRs of 37–48-month-old children (68 % EER). The supply of the other minerals sometimes considerably diverged from the recommended allowance, depending on the element and the children’s age and sex. In total, the supply of the following elements exceeded the RDA: Mg (105–216 %), P (114–156 %), Zn (97.2–190 %) and Cu (137.5–212 %). The consumption of Mg in the group of boys aged 25–36 months (133 % RDA) was 39 % lower than in the group of boys aged 12–24 months (185 % RDA) and about 62 % lower than in the same age group of girls (216 % RDA). Neither the children’s sex nor age had influence on the intake of P and Zn in the DFR. The content of Cu in the group of boys aged 25–36 months was lower than in the groups aged 12–24 months and 37–48 months by 25 and 23 % (140 vs 155 and 205 % RDA), respectively. On the other hand, the supply of Ca and K was lower than the RDA and it reached 44–75 % for Ca and 48–72 % for K. The consumption of Ca was 29 % lower in the group of boys aged 25–36 months than in the group of younger boys (12–24 months), and it amounted to 49 and 69 % of the RDA, respectively. Apart from that, the DFRs were also characterised by too low Ca/P molar ratio, which ranged from 0.5 to 0.7. The value of the ratio was lower in the diet of the children aged 37–48 months (0.47) than in the groups aged 12–24 months (0.69) and 25–36 months (0.55). The supply of Fe varied in different age groups and ranged from 57.0 to 117.2 %, while there were significant age-dependent and sex-dependent differences in the consumption of this element, i.e. 12–24-month-olds (117.2 % RDA) and 37–48-month-olds (57.0 % RDA). The content of this element in the DFR was significantly higher in the girls and in the boys aged 25–36 months, i.e. 114.3 RDA vs 60.3 % RDA.

**Table 1:** The characteristics of the group under study

	12–24 months			25–36 months			37–48 months		
	Total	Girls	Boys	Total	Girls	Boys	Total	Girls	Boys
*N*	27	9	18	19	12	7	29	18	11
Body weight [kg]^*^	12.1 ± 2.0^a^	11.2 ± 1.4	12.5 ± 2.1	14.0 ± 1.8^b^	14.3 ± 1.5	13.5 ± 2.3	15.2 ± 2.2^c^	14.8 ± 2.3	15.9 ± 2.0
Height [cm]^*^	85.6 ± 3.9^a^	84.6 ± 4.5	86.2 ± 3.5	94.4 ± 4.0^b^	94.4 ± 3.2	94.5 ± 5.4	98.7 ± 4.3^c^	98.9 ± 3.2	98.3 ± 5.3
LA	1.3 ± 1.2^b^	1.6 ± 1.4	1.1 ± 1.0	1.3 ± 1.0^a^	1.3 ± 0.9	1.3 ± 1.2	0.0 ± 0.8^a^	0.1 ± 0.8	−0.1 ± 0.9
WA^*^	0.9 ± 0.8^b^	1.0 ± 0.7	0.9 ± 0.9	1.0 ± 1.0^b^	1.2 ± 0.8	0.6 ± 0.9	0.1 ± 1.0^a^	−0.1 ± 1.0	0.4 ± 0.9
BMI	16.4 ± 2.6	15.7 ± 2.0	16.8 ± 2.8	15.6 ± 1.2	15.8 ± 1.3	15.3 ± 0.9	15.6 ± 1.8	15.1 ± 2.0	15.1 ± 2.0
BMI z-score*	0.1 ± 1.6	−0.3 ± 1.7	0.3 ± 1.6	0.0 ± 1.1	0.3 ± 1.1	−0.6 ± 0.9	0.0 ± 1.4	−0.3 ± 1.6	0.7 ± 0.9

^*(a,b,c)^Statistically significant differences between age groups (*p* < 0.01)

BMI z-score* body mass index-for-age, *LA* length/height-for-age, *WA* weight-for-age

**Table 2:** The average daily consumption of energy and minerals in the DFR of children aged 12–48 months depending on their age and sex

Index		Age (months)					
		12–24		25–36		37–48	
		Boys	Girls	Boys	Girls	Boys	Girls
Energy [kcal/day]	Mean ± SD	1061.85 ± 206.71	946.91 ± 229.49	967.83 ± 265.73	1035.08 ± 187.12	953.90 ± 264.86	950.42 ± 264.86
	% EER	106.2	94.7	96.8	103.5	68.1	67.9
Ca [mg/day]	Mean ± SD	484.75 ± 211.50^b^	439.8 ± 123.95	344.95 ± 64.68* ^a^	535.82 ± 169.39*	461.82 ± 248.93^ab^	400.34 ± 138.60
	% RDA	69.3	62.8	49.3	76.6	46.1	40.3
P [mg/day]	Mean ± SD	680.29 ± 190.91	526.30 ± 194.37	651.00 ± 154.36	717.23 ± 167.20	639.58 ± 269.89	633.38 ± 157.95
	% RDA	147.9	114.4	141.5	155.9	127.9	126,7
Ca/P	Mean ± SD	0.53 ± 0.17	0.69 ± 0.30^B^	0.42 ± 0.15	0.55 ± 0.14^B^	0.54 ± 0.18	0.47 ± 0.09^A^
Mg [mg/day]	Mean ± SD	147.73 ± 38.13^b^	123.86 ± 54.27	106.25 ± 28.87* ^a^	172.72 ± 72.26*	142.23 ± 59.18^ab^	127.33 ± 37.17
	% RDA	184.6	154.8	132.8	215.9	109.0	97.9
Na [mg/day]	Mean ± SD	856.04 ± 326.30	769.38 ± 415.37	1216.98 ± 1000.87	769.38 ± 415.37	806.77 ± 416.83	763.00 ± 160.85
	% AI	141.1	102.6	162.3	109.4	80.7	76.3
K [mg/day]	Mean ± SD	1732.33 ± 512.69	1403.41 ± 434.59	1326.00 ± 450.46	1690.64 ± 345.38	1491.80 ± 411.24	1509.18 ± 304.36
	% AI	72.2	58.5	55.3	70.4	48.1	48.7
Fe [mg/day]	Mean ± SD	8.17 ± 3.42^b^	8.30 ± 0.81^B^	4.22 ± 0.75* ^a^	8.00 ± 3.31* ^B^	5.47 ± 1.68^a^	6.0 ± 1.91^A^
	% RDA	116.7	118.5	60.3	114.3	54.7	60.0
Zn [mg/day]	Mean ± SD	5.77 ± 1.69	5.30 ± 1.04	4.95 ± 1.47	5.78 ± 1.75	4.94 ± 1.80	4.86 ± 1.22
	% RDA	192.4	176.7	164.8	192.7	98.8	97.2
Cu [mg/day]	Mean ± SD	0.64 ± 0.20^b^	0.54 ± 0.11	0.48 ± 0.08^a^	0.85 ± 0.80	0.62 ± 0.22 ^b^	0.55 ± 0.17
	% RDA	212.2	181.5	140.5	284.9	155.0	137.5

*DFR* daily feed ratio, *EER* energy efficiency rating, *RDA* recommended dietary allowance, *AI* adequate intake

*Sex-dependent differences within an age group (*p* < 0.05)

^a, b^Age-dependent differences among boys (*p* < 0.05)

^A, B^Age-dependent differences among girls (*p* < 0.05)

**Table 3:** The average daily consumption of energy and minerals in the DFR of children aged 12–48 months depending on their age

Index		Age (months)		
		12–24	24–36	37–48
		Total	Total	Total
Energy [kcal/day]	Mean ± SD	1017.74 ± 219.42	1009.87 ± 213.72	951.73 ± 266.88
	% EER	101.7	100.9	67.9
Ca [mg/day]	Mean ± SD	471.12 ± 187.44	464.31 ± 166.52	435.93 ± 207.05
	% RDA	67.3	66.3	43.59
P [mg/day]	Mean ± SD	633.42 ± 201.00	692.39 ± 160.66	636.97 ± 224.01
	% RDA	137.7	150.5	127.4
Ca/P	Mean ± SD	0.58 ± 0.22	0.51 ± 0.15	0.51 ± 0.15
Mg [mg/day]	Mean ± SD	140.47 ± 43.83	147.79 ± 67.19	135.96 ± 50.40
	% RDA	175.6	184.7	104.6
Na [mg/day]	Mean ± SD	829.66 ± 348.29	969.36 ± 661.96	788.34 ± 327.23
	% AI	110.6	129.2	78.8
K [mg/day]	Mean ± SD	1632.23 ± 504.65	1553.90 ± 415.27	1499.12 ± 360.64
	% AI	68.0	64.7	48.4
Fe [mg/day]	Mean ± SD	8.2 ± 2.86^b^	6.58 ± 3.22^ab^	5.70 ± 1.75^a^
	% RDA	117.2	94.1	57.0
Zn [mg/day]	Mean ± SD	5.63 ± 1.52	5.47 ± 1.65	4.90 ± 1.55
	% RDA	187.7	182.3	98.0
Cu [mg/day]	Mean ± SD	0.61 ± 0.18	0.69 ± 0.65	0.59 ± 0.20
	% RDA	202.9	230.8	147.5

*DFR* daily feed ratio, *EER* energy efficiency rating, *RDA* recommended dietary allowance, *AI* adequate intake

^a, b^Age-dependent differences for the whole group (*p* < 0.05)

Tables 4 and 5 show the data on the content of selected minerals in the children’s hair, depending on their age and sex. The statistical analysis revealed that the content of Ca in the hair of the boys aged 25–36 months was 27 % lower than in the younger group (12–24 months). The concentration of Zn in the hair of the children aged 25–36 months was 26 % higher than in the oldest group (166.26 vs 131.54 μg/g). As far as the content of Mg and Fe in the children’s hair is concerned, there were no significant differences observed between the age groups or sexes. The content of Zn in the older girls’ hair (37–48 months) was respectively 41 and 32 % lower than in the hair of the girls aged 12–24 and 25–36 months. The content of Cu was comparable in all the age groups. However, the content of this element in the hair of the girls aged 12–24 and 25–36 months was higher than in the hair of the boys from the same age groups by 51 and 58 %, respectively. Table 6 shows the data with the occurrence of significant correlations. The analysis of correlations proved the presence of dependence between the content of Ca and Mg in the hair of the children aged 12–24 months (*r* = 0.43) and 37–48 months (*r* = 0.57) and between the content of Zn and Ca (*r* = 0.75) and Mg (*r* = 0.64) in the hair of the children aged 25–36 months.

**Table 4:** The content of minerals (Cu, Zn, Fe, Ca, Mg [μg/g]) in the hair depending on the age and sex

Index		Age (months)					
		12–24		25–36		37–48	
		Boys	Girls	Boys	Girls	Boys	Girls
Ca [μg/g]	Mean ± SD	501.12 ± 187.64^b^	510.02 ± 151.88	366.07 ± 86.04^a^	480.80 ± 214.36	494.90 ± 248.77^ab^	485.29 ± 262.12
Mg [μg/g]	Mean ± SD	22.13 ± 8.22	27.00 ± 10.34	23.89 ± 7.93	25.46 ± 8.15	21.58 ± 7.37	29.52 ± 13.56
Fe [μg/g]	Mean ± SD	24.48 ± 5.96	30.64 ± 17.46	29.15 ± 7.89	32.66 ± 6.81	22.97 ± 8.52	28.60 ± 10.99
Zn [μg/g]	Mean ± SD	147.01 ± 51.61	178.80 ± 47.22^B^	162.88 ± 49.45	168.41 ± 36.59^B^	139.89 ± 26.72	126.77 ± 43.91^A^
Cu [μg/g]	Mean ± SD	9.16 ± 3.52*	13.84 ± 4.52*	7.24 ± 1.18*	11.45 ± 6.51*	8.81 ± 2.58	9.26 ± 2.40

*Sex-dependent differences within an age group (*p* < 0.05)

^a, b^Age-dependent differences among boys (*p* < 0.05)

^A, B^Age-dependent differences among girls (*p* < 0.05)

**Table 5:** The content of minerals (Cu, Zn, Fe, Ca, Mg [μg/g]) in the hair depending on the age

Index		Age (months)		
		12–24	25–36	37–48
		Total	Total	Total
Ca [μg/g]	Mean ± SD	503.14 ± 176.73	436.67 ± 180.69	488.89 ± 251.74
Mg [μg/g]	Mean ± SD	23.68 ± 8.99	25.01 ± 7.81	27.00 ± 12.11
Fe [μg/g]	Mean ± SD	25.85 ± 8.50	31.78 ± 6.68	26.86 ± 10.29
Zn [μg/g]	Mean ± SD	156.68 ± 51.45^ab^	166.26 ± 40.72^b^	131.54 ± 38.38^a^
Cu [μg/g]	Mean ± SD	10.72 ± 4.53	9.65 ± 3.93	9.10 ± 2.43

^a, b^Age-dependent differences for the whole group (*p* < 0.05)

**Table 6:** Significant correlations between the energy intake, the consumption of individual minerals in the DFR and their content in the hair and between individual bioelements in the children’s hair depending on their age

Age	Significant correlations			
	Between energy intake, consumption of minerals in DFR and their content in children’s hair		Between individual bioelements in children’s hair	
12–24 months	kcal/Fe Ca/Zn Na/Fe Ca/Ca P/Ca	−0.81 −0.65 −0.75 0.92 0.48	Mg/Cu Ca/Fe Ca/Mg	0.42 −0.47 0.43
25–36 months	Ca/Ca P/Ca	0.99 0.50	Cu/Fe Zn/Ca Zn/Mg	0.72 0.75 0.64
37–48 months	kcal/Ca Cu/Ca Zn/Ca Mg/Ca Ca/Ca P/Ca	0.70 0.64 0.64 0.75 0.90 0.80	Cu/Mg Ca/Mg	0.51 0.57

*DFR* daily feed ratio

Apart from that, the research revealed a relatively high correlation (0.90–0.99) between the supply of Ca in the DFR and its content in the children’s hair in all the age groups.

## Discussion

The correct balance of minerals plays an important role in both humans and animals [7]. A long-lasting deficiency, excess or abnormal ratio of inorganic nutrients may cause serious consequences in the form of specific diseases or nutrition disorders contributing to diseases of civilisation, such as atherosclerosis, osteoporosis, cancer and diabetes [3]. Due to the role of minerals in metabolism and the body’s inability to synthesise them, adequate quantities of these essential food components should be provided. This study assessed the intake of energy and minerals in the DFR and their influence on the content of minerals in the hair of 1–4-year-old children staying at home or attending a nursery school. The analysis of DFRs for the children aged 1–4 years showed that there was not enough Ca in the diet. Dymkowska-Malesa and Szparaga [8] and Sochacka-Tatara et al. [9] observed that the supply of Ca in food rations in nursery schools was too low and on average it reached 80 % of the RDA. Uush [10] observed that 1–3-year-old Mongolian children consumed 39 % less Ca in their DFRs than the recommended value. The deficit of Ca was also observed in the diets of Indian and Brazilian children [11, 12]. Low supply of Ca combined with low supply of vitamin D may cause disordered Ca homeostasis and irregular bone development [13].

At present, it is thought that in order to guarantee the optimal calcium absorption, the calcium/phosphorus molar ratio in the diet should be 1:1, or 1.3:1 in weight units [14]. The Ca/P molar ratio in daily food rations was inadequate in all the age groups, and it amounted to about 0.46. Mina et al. [15] observed that the Ca/P molar ratio in the diets of healthy children was slightly higher than in the diets of 4–12-year-old children with celiac disease (0.728 vs 0.606).

There was high content of Mg in the food rations under study. It exceeded the 1–4-year-old children’s demand for the element by about 75 %. Dymkowska-Malesa and Szparaga [8] analysed food rations in nursery schools, and they also proved that the supply of Mg was 225 % of the RDA. Shibata et al. [16] found that there was an excessive amount of Mg (104–115 mg/d) in 3–4-year-old Japanese children’s DFRs. The analysis of menus in a vegetarian nursery school also revealed excessive amounts of this element in the diet [17]. It is thought that a diet with excessive supply of Mg may disturb the right proportions between Ca and Mg, i.e. 2:1. However, on the other hand, it is necessary to stress the fact that the absorption of magnesium from the diet may be limited by the presence of antinutrients [18].

This research did not reveal differences in the consumption of Na and K among 1–4-year-old children. However, the consumption of Na was too high, whereas the consumption of K was too low in the population under study. Tian et al. [19] obtained similar results in their research on children aged 1–3 and 4–5 years. Frank et al. [20] observed that the intake of Na and K by 6-month-old children was respectively four and two times lower than the intake by 4-year-old children.

In general, the supply of Fe in the DFR was adequate, except for a group of boys aged 25–36 months, whose diet provided only about 60 % of the recommended amount. Grajeta et al. [21] and Niinikoski et al. [22] observed the adequate supply of Fe in daily food rations. Eussen et al. [23] studied the literature and observed that in most countries, the average consumption of iron in the diets of children aged 6–12 and 12–36 months was close to the RDA. It is necessary to remember that small children are particularly endangered to the consequences of iron deficit due to the rapid development of their brains. Iron is necessary as a cofactor of many enzymes. If the insufficient consumption of Fe continues, it may cause anaemia, which has particularly negative consequences to pre-school children. Low haemoglobin decreases physical capacity, causes malfunction of the immune system and simultaneously affects children’s cognitive functions and their learning processes.

The consumption of Zn in the DFRs under study ranged from 4.86 to 5.78 mg/day, and it was only slightly lower than the values of Zn consumption which were observed by Schrey et al. [24] (5.7 mg/day), who analysed DFRs of German children aged 1.5–5.3 years. Studies on American children proved that the average daily consumption of Zn by children aged <1, 1–3 and 4–5 years was 6.6, 7.6 and 9.1 mg/day, respectively. The element was rarely deficient, i.e. only in 1 % of DFRs [25]. Brown et al. [26] proved that the adequate supply of Zn positively affected children’s somatic development. Bhutta et al. [27] observed that the adequate supply of Zn decreased the occurrence and intensity of children’s infections. Muller et al. [28] observed that the supplementation of Zn did not affect the development/height of malnourished children from West Africa.

Like in this study, Schrey et al. [24] analysed the DFRs of children aged between 1.5 and 5.3 years. They observed that the average Cu consumption fully satisfied the demand for this element. Goshima et al. [29] also did not observe Cu deficits in the DFRs of children aged 3–5 years.

This study showed that the intake of energy and minerals in children aged 37–48 months was lower than in the younger groups. It was caused by the high share of enriched food in the children’s diet, including food products for special nutritional purposes for infants and small children. Also, the nursery school children were observed to eat only part of the allocated food rations.

Inadequate supply of minerals in DFRs (low content of Ca and K, excessive content of Mg, P, Na, Zn and Cu) is caused by improper combinations of products in the children’s diets. This situation results from the fact that parents and the people in charge of children’s nutrition in nursery schools do not have sufficient nutritional knowledge. These people’s ignorance causes the development of children’s improper nutritional habits [8, 30]. The results of this study indicate the need to educate parents and the people in charge of children’s nutrition in nursery schools so they know how to feed the youngest children well.

This study revealed significant differences in the content of individual bioelements, depending on the children’s sex and age. The available publications do not provide much data on the content of minerals in the hair of healthy children aged up to 4 years. Most publications describe children with health problems. Heavy metals were the elements whose content was usually measured in children’s hair (Pb, Cd, Hg) [31, 32].

The content of minerals in the hair depends on many factors, including physiological changes in the human organism, absorption and excretion capacity, activity of the endocrine system, sex-dependent metabolism, changes in the hair structure, environmental exposure, the state of health, stress and diet. In this study, the average contents of Ca, Mg, Fe, Zn and Cu were 476.23, 25.23, 28.16, 151.49 and 9.82 μg/g, respectively. On the other hand, Kolarzyk et al. [33] measured the content of Ca, Mg, Fe, Zn and Cu in nursery school children’s hair and noted the following values: 296.00, 44.63, 16.2, 152.30 and 11.59 μg/g, respectively. Wójciak et al. showed that the content of Ca in the hair of obese children was higher in the group of girls than in the group of boys (839.1 vs 596.1 μg/g) [34]. However, this study did not reveal sex-dependent influence on the content of Ca in the children’s hair. Gürgöze et al. [35] observed that the content of Fe (5.9 vs 12.9 μmol/l) and Zn (16 vs 19.1 μmol/l) in the hair of 1–4-year-old children with anaemia was much lower, whereas the content of Cu (25.1 vs 21.9 μmol/l) was higher in healthy children at the same age. Park et al. [36] noted that the concentration of Zn in the hair of infants who had been weaned was not caused by the deficit of Fe and it decreased as the children grew older (*r* = 0.25). Kemmer et al. [37] observed that about 17 % of children aged 6–60 months living in refugee camps had low content of Zn in their hair. According to these authors, when the content of Zn in the hair is <70 μg/g, it indicates the deficit of this element in the diet. In this study, we did not observe such low values of Zn content in the children’s hair. Kauf et al. [38] measured the content of Zn in the hair of infants aged up to 478 days and noted a value of 212 μg/g. The researchers did not observe the dependence between the content of bioelements in the infants’ hair and their sex. The concentration of Zn in the hair of children aged 11–24 months living in rural areas in Brazil was 134.3 μg/g [39]. The research by Kozielec et al. [40] revealed that the content of Cu in the hair of children aged 1, 2 and 3 years was similar to the values observed in this study and it reached 11.52, 9.1 and 8.7 μg/g, respectively. Sakai et al. [41] observed that the mean concentrations of copper in hair was significantly higher in the male group than in the female group. This relation was different in our study.

In this study, we observed only a correlation between the consumption and concentration of Ca in hair. The absence of correlations between the consumption of other minerals and their concentration in hair may have been caused by different absorption of nutrients in the body. Minerals are absorbed from the diet to a different extent, depending on the type of element, the state of saturation of the body and interaction with other bioelements. Also, the food rate of release is different, depending on the food product and different conditions prevailing in different sections of the gastrointestinal tract. Also, the absence of correlations may have been caused by imprecise evaluation of individual microelement intake with the Dietetic 2 software (under- or overestimation of real intake) and a short-term dietary record in comparison with the duration of hair growth, individual hair growth rate as well as different biological roles of minerals, their absorption rates and factors determining the deposition of minerals in the hair matrix. Kolarzyk et al. [33] analysed the concentration of minerals in the hair of children aged 4.5–7.5 years with reference to the supply of these elements in the DFR. They observed that excesses and deficiencies of minerals in the hair were not strictly related to inadequate nutrition. Inadequate amounts of minerals were observed both in underweight and overweight children (BMI assessed) as well as in children with excessive (above 95 percentile) and deficient (below 5 percentile) percentage of body fat.

As far as Ca and Fe are concerned, although their supply in the DFR was higher, their concentrations in the hair were lower than in this study. Senofonte et al. [42] observed that the concentrations of Ca, Mg, Fe, Zn and Cu tended to increase with age. According to Długaszek et al. [43], this tendency can be observed up to the age of about 40 and it does not apply to Fe.

In this study, we observed correlations between the content of Ca in the hair of children aged 1–4 years and the intake of energy, Ca and Zn. The study by Kayakırılmaz [44] revealed significant positive correlations between the consumption of Ca, Mg and Cu in the DFR and their concentrations in pre-school children’s hair. Chojnacka et al. [45] observed that feeding habits influenced the content of bioelements in the hair. On the other hand, Suliburska [46] found that the content of Ca, Mg, Fe and Zn in the DFR of women at the age of fertility was negatively correlated with the content of Cu in their hair. Lahti-Koski et al. [47] observed that the consumption of Fe in the DFR was not related to its content in the hair. Park and Shin [48] proved that pre-school children’s intake of Zn did not affect the concentration of this element in the hair. Similarly, Gonzalez-Munoz et al. [49] observed that the content of minerals in the hair did not correspond to their intake in the DFR.

The analysis of correlations proved the presence of dependence between the content of Ca and Mg in the hair of the children aged 12–24 months (*r* = 0.43) and 37–48 months (*r* = 0.57) and between the content of Mg and Zn (*r* = 0.64) in the hair of the children aged 25–36 months. The Ca/Mg and Ca/Zn correlation coefficients in the hair of girls aged 1–10 years were 0.81 and 0.48, respectively [43]. Jeruszka-Bielak and Brzozowska [50] observed the presence of a Ca/Mg correlation in young women’s hair (*r* = 0.57). The result was similar in the study by Dunicz-Sokołowska et al. [51] (*r* = 0.7).

As we observed in this study, neither the children’s sex nor age had influence on the energy intake, the content of minerals in the DFR or in the hair. The children’s age affected the consumption of Ca, Mg, Fe and Cu in the DFR and the content of Ca and Zn in the hair. On the other hand, the children’s sex affected the intake of Ca, Mg and Fe in the DFR and the content of Cu in the hair.

## Results

1. The daily food rations of the children aged 12–36 months were characterised by adequate energy value, but the values of Ca and K were too low, whereas the content of Mg, Zn and Cu was high. On the other hand, the DFRs of the children aged 37–48 months provided sufficient amounts of Mg and Zn, but the value of Cu was too high, whereas energy, Ca, K and Fe values were too low.
2. The children aged 37–48 months were characterised by much lower WA and LA than the younger children. The nutritional inadequacy had impact on the children’s development.
3. About 42.7 % of the children under study were characterised by an abnormal state of nutrition.
4. There are significant correlations (*r* > 0.9) between the supply of Ca in the diet of children aged 1–4 years and the content of this element in their hair.
5. An inadequately balanced diet needs to be corrected by educating parents or guardians in appropriate nutrition.
